# Effect of Preparation Methods on Crystallization Behavior and Tensile Strength of Poly(vinylidene fluoride) Membranes

**DOI:** 10.3390/membranes3040389

**Published:** 2013-11-21

**Authors:** Jie Liu, Xiaolong Lu, Chunrui Wu

**Affiliations:** State Key Laboratory of Hollow Fiber Membrane Materials and Membrane Processes, Institute of Biological and Chemical Engineering, Tianjin Polytechnic University, Tianjin 300387, China; E-Mail: liujie198728@163.com

**Keywords:** poly(vinylidene fluoride) membranes, preparation methods, crystallization, tensile strength

## Abstract

Poly(vinylidene fluoride) (PVDF) membranes were prepared by non solvent induced phase separation (NIPS), melt spinning and the solution-cast method. The effect of preparation methods with different membrane formation mechanisms on crystallization behavior and tensile strength of PVDF membranes was investigated. Fourier transform infrared spectroscopy-attenuated total reflectance (FTIR-ATR) and X-ray diffraction (XRD) were employed to examine the crystal form of the surface layers and the overall membranes, respectively. Spherulite morphologies and thermal behavior of the membranes were studied by polarized light optical microscopy (PLO) and differential scanning calorimetry (DSC) separately. It was found that the crystallization behavior of PVDF membranes was closely related to the preparation methods. For membranes prepared by the NIPS method, the skin layers had a mixture of α and β phases, the overall membranes were predominantly α phase, and the total crystallinity was 60.0% with no spherulite. For melt spinning membranes, the surface layers also showed a mixture of α and β phases, the overall membranes were predominantly α phase. The total crystallinity was 48.7% with perfect spherulites. Whereas the crystallization behavior of solution-cast membranes was related to the evaporation temperature and the additive, when the evaporation temperature was 140 °C with a soluble additive in the dope solution, obvious spherulites appeared. The crystalline morphology of PVDF exerted a great influence on the tensile strength of the membranes, which was much higher with perfect spherulites.

## 1. Introduction

Poly(vinylidene fluoride) (PVDF) as a semi-crystalline polymer, is widely used as a commercial polymeric membrane material because of its outstanding properties: excellent chemical resistance, thermal stability and high mechanical strength [1,2,3,4,5]. It is well-known that PVDF has four crystalline forms: the nonpolar α form and the polar β, γ and δ forms [6,7]. The most common polymorph is the nonpolar α phase with a chain conformation of trans-gauche (TG^+^TG^−^). The polar β phase has an all trans-planar zigzag conformation (TTT) [8,9]. Conversion between the distinct PVDF phases may occur through convenient thermal and mechanical treatments [10,11].

In recent years, lots of investigations have been focused on controlling the polymorph of PVDF and also, improving the performance of the existing PVDF membranes in terms of anti-fouling properties, mechanical strength and chemical resistance. Many valuable results about the relationship between fabrication conditions and PVDF polymorph have been obtained. The research results mainly include several aspects of the following: (1) effect of preparation conditions on the total crystallinity and polymorph of non solvent induced phase separation (NIPS) membranes [12,13,14,15,16,17,18]; (2) melt crystallization of PVDF [19,20,21]; (3) solution-cast membranes [6,22,23]; (4) influence of stretching on crystalline phase structure and morphology of PVDF membranes [10,24,25,26,27]. However, comprehensive discussion on the influence of the preparation methods on the crystallization behavior and tensile strength of PVDF membranes has seldom been reported and little is known on how to control the crystalline structure of PVDF membranes through convenient choice of the preparation conditions.

In general, tensile strength is one of the key factors that influence the industrial application of PVDF membranes. In addition, crystalline conformation and total crystallinity of PVDF are very important in determining the tensile strength of membranes. Crystalline conformation can be converted from one form to the other by certain treatments. Buonomenna *et al*. [16] proved that the percentage of the α phase was very important in obtaining PVDF membranes with a higher mechanical strength; the predominance of α phase crystallites contributed to improving its mechanical properties. The degree of crystallinity of PVDF can range between 35% and 70%, and crystallization of PVDF is controlled by a number of variables including molecular weight, molecular weight distribution, polymerization method, thermal history and cooling rates [28]. Mohajir *et al*. [8,9] found that the mechanical properties of PVDF could be improved by increasing the total crystallinity. At the same time, there are great differences in mechanical properties of PVDF membranes prepared by different methods.

In this study, PVDF membranes were prepared through NIPS, melt spinning and the solution-cast method. The effects of the preparation methods with different membrane formation mechanisms on the crystallization behavior and tensile strength of PVDF membranes were investigated. The objective of the current work was to investigate the differences in tensile strength of PVDF membranes prepared by various methods, which can help to control the crystalline structure and improve the tensile strength of existing PVDF membranes.

## 2. Experimental Section

### 2.1. Materials

The PVDF resin used was SOLEF 6010 from Solvay Solexis Company (Brussels, Belgium), which has a melt flow index of 6 g/10 min (230 °C, 5 kg). The crystallizing point (crystallizing point is the onset of polymer melting) and melting temperature of the as-received resin are 138 and 173 °C according to the product introduction. Industrial grade dimethylacetamide (DMAc) was used as solvent without further purification, and it was purchased from Samsung Company (Seongnam, Korea) with a boiling point of 166 °C.

### 2.2. Preparation of NIPS Membranes

A mixture of PVDF/DMAc/PG (16/62/22 wt %) was dissolved in a glass flask at 70 °C, followed by stirring until the solution became homogeneous. PG is a self-made additive (a mixture of a certain proportion of n-propanol, n-hexanol and glycerol), which favors the demixing process. The dope solution was then transferred into a tank, kept at a constant temperature of 70 °C for 6 h to eliminate air bubbles in the solution. Finally, the hollow fiber membranes were prepared by the NIPS method using a tube-in orifice spinneret, membranes prepared as such were the same as the membranes in our previous work [29]. The casting solution and bore fluid passed through the orifice and inner tube, respectively. The bore fluid and coagulant bath were both ultrafiltrated (UF) water with temperatures of 70 and 50 °C, respectively. The nascent membranes were taken up at a drawing rate of 25 m/min and immersed in UF water for 48 h to remove the residual DMAc, then kept in an aqueous glycerol solution with specific gravity of 1.08 for 48 h to prevent the collapse of the porous structures. The membranes were dried finally in ambient air. The inner diameter and wall thickness of NIPS hollow fiber membranes is 850 and 180 µm, respectively. The flat sheet membranes with a thickness of approximately 17 µm were prepared by a glass blade with clean glass, and the coagulant bath was 50 °C UF water with no residence time in air.

### 2.3. Preparation of Melt Spinning Membranes

A twin-screw extruder was used for the preparation of melt spinning hollow fiber membranes. Melt spinning PVDF hollow fiber membranes were melt-spun at 230 °C through a tube-in orifice spinneret with inner and outer diameters of 3.6 and 5.2 mm. First, the melts were extruded out of the spinneret at 230 °C by using air as bore gas. Then, the hollow fiber membranes were immersed in 25 °C UF water with a drawing rate of 70 m/min. The inner diameter and wall thickness of the melt spinning hollow fiber membranes is 900 and 115 µm, respectively. The flat sheet membranes with thickness of approximate 6 µm were prepared on a hot plate at 230 °C, then cooled at the rate of 50 °C/min.

### 2.4. Preparation of Solution-Cast Membranes

PVDF was dissolved in DMAc at 60 °C under continuous agitation using a magnetic stirrer in a hermetically sealed glass flask until completely dissolved. The dope composition was varied by adding a self-made additive PG. After air bubbles were removed completely, the dope solution was spread onto a hot plate by a glass blade, and two types of membranes with thicknesses of 6 and 45 µm were obtained by evaporating the dope solution separately for 1 h at a special temperature. The evaporation time of 1 h was sufficient for evaporation of almost all the solvent and formation of sustainable membranes. The membranes were removed from the hot plate by immersing into a 30 °C water bath, and kept in an oven with a temperature of 60 °C for 8 h to remove possible solvent residues. The dope composition and preparation conditions for various membranes are listed in Table 1.

**Table 1:** Preparation conditions for solution-cast membranes.

Membrane label	PVDF concentration in casting solution (wt %)	PG concentration in casting solution (wt %)	Evaporation temperature (°C)
F5-60	5	0	60
F5-140	5	0	140
F5-7-60	5	7	60
F5-7-140	5	7	140

Notes: PVDF = Poly(vinylidene fluoride); PG is a self-made additive (a mixture of a certain proportion of n-propanol, n-hexanol and glycerol).

In the solution-cast process, the evaporation temperatures were 60 °C and 140 °C. These two temperatures were chosen because of their different evaporation rates and their position in relation to the crystallizing point of PVDF (crystallizing point was the onset of polymer melting [23], when the evaporation temperature was 140 °C, which was higher than the crystallizing point 138 °C, both precipitation crystallization and melt crystallization were able to occur, whereas at 60 °C only precipitation crystallization occurred).

### 2.5. Membrane Characterization

Infrared spectra collected directly from the outer membrane surface were obtained using a Fourier transform infrared spectroscopy (FTIR) spectrophotometer (Bruker Tensor 37, Karlsruhe, Germany) in the attenuated total reflection (ATR) mode. The penetration depth is typically less than one micron and thus the ATR spectra give a good representation of the skin of the membrane. From the infrared spectra of samples in the wave-number range between 700 and 1500 cm^−1^, the crystalline phase of PVDF can be verified. The α and β phases of PVDF are associated with the vibration band peaks at 764 and 840 cm^−1^, respectively [26].

The degree of crystallinity of α and β phases on the surface of the membrane can be determined by comparing the absorbance of vibration band peaks of the samples at 764 and 840 cm^−1^, based on the Beer-Lambert law:

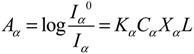
(1)

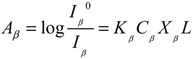
(2)
where, *A_α_* and *A_β_* are the absorbencies, *I*^0^ and *I* are the incident and transmitted optical intensities, respectively. *K* is the absorptivity at the respective wavenumber, and *X* is the crystallinity of each phase. The values of *K_α_* and *K_β_* are equal to 6.1 × 10^4^ and 7.7 × 10^4^ cm^2^/mol. *C_α_* and *C_β_* are calculated from the densities of the two phases, resulting in values of 0.0301 and 0.0308 mol/cm^3^, respectively [20,30]. 

Crystalline structures of the overall membranes were investigated with an X-ray Diffractometer (Bruker D8 advance, Karlsruhe, Germany), using Cu *K*_α_ radiation at a voltage of 40 kV and a current of 40 mA. Intensities were measured in the range 10° < 2θ < 45°, typically with step scans of 0.05°. The characteristic diffraction peas of α and β phases were at 2θ (17.8°, 18.3°, 19.9° and 26.7°) and 2θ (20.6°), respectively [16]. 

The melting temperature and total crystallinity of the PVDF membranes was characterized by differential scanning calorimeter (Perkin-Elmer DSC-7, Wellesley, MA, USA). The heating rate was set to 10 °C/min.

The PVDF total crystallinity *X_c_* was calculated by [31]:

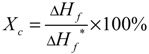
(3)
where *∆H_f_* ^*^ = 104.7 J/g, is the melting enthalpy for a 100% crystalline PVDF, *∆H_f_* is the melting enthalpy of PVDF membranes measured in DSC.

Spherulite morphologies were observed by polarized light optical microscopy (Olympus BX51, Tokyo, Japan).

Tensile strength of PVDF membranes was measured by an Electronic Single-yarn Tensile Tester (room temperature, 500 mm/min). The tensile strength was calculated by:

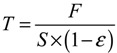
(4)
where *T* is the tensile strength, Pa; *F* is the breaking strength, N; *S* is the cross section area of the membrane, m^2^; *ε* is the porosity of the membrane, %.

## 3. Results and Discussion

### 3.1. Crystalline Structures in the Surface Layers

**Figure 1:**
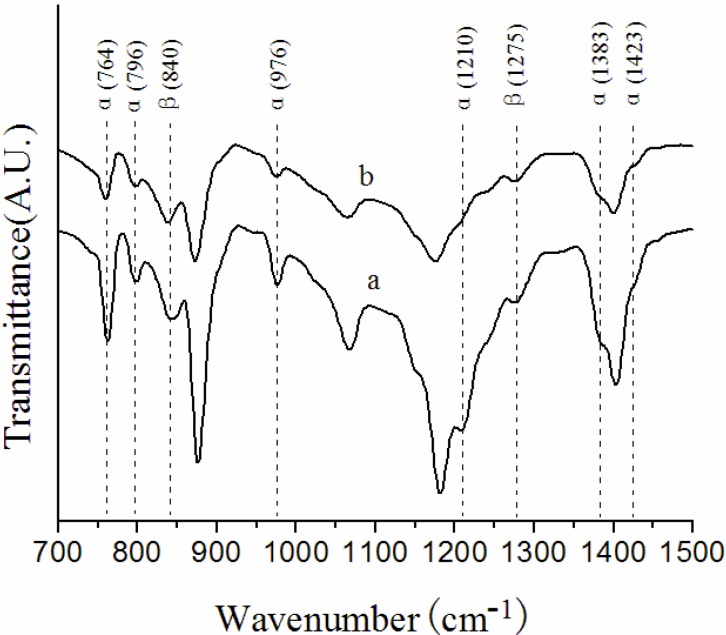
Fourier transform infrared spectroscopy-attenuated total reflectance (FTIR-ATR) spectra of (**a**) non solvent induced phase separation (NIPS) membrane; (**b**) melt spinning membrane.

**Figure 2:**
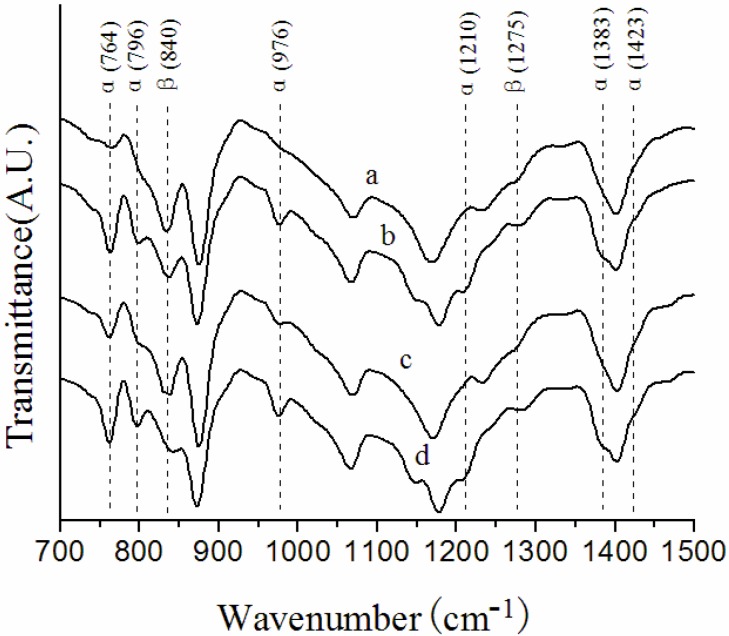
FTIR-ATR spectra of solution-cast membranes prepared at different conditions. (**a**) F5-60; (**b**) F5-140; (**c**) F5-7-60; (**d**) F5-7-140.

Figure 1 and Figure 2 show the FTIR-ATR spectra of the surface layers of the NIPS membrane, melt spinning membrane and solution-cast membranes prepared at different conditions. All the membranes have a mixture of α and β phases in the outer skin. The characteristic bands of α and β phases are indicated in the two figures, and α/β values are summarized in Table 2.

As shown in Figure 1 and Table 2, the α/β value of the NIPS membrane is 1.30, however, the characteristic bands of the α phase become extremely weak and the α/β value decreases to 0.88 in the melt spinning membrane, suggesting that the β phase increases a lot in the surface layers of melt spinning membranes. An explanation for this phenomenon might be given on considering that the dope solution extruded out of the spinneret was immersed into a 50 °C water bath after a 3 cm air bath in the preparation of the NIPS membrane, the curing rate of the outer surface was too fast, consequently, the crystallization rate of PVDF increased. While the melt spinning dope solution extruded out at 230 °C went through a longer air bath of 50 cm, the curing rate of the outer surface was slowed down, thus, resulting in a slower crystallization rate of PVDF that favored the formation of the β phase. According to previous study [14], the increase in polymer concentration could preferably lead to better oriented packing of CH_2_–CF_2_ dipoles (TTT conformation) in the surface, thus effectively cutting the time required for the onset of β phase nucleation. The PVDF concentration of the melt spinning dope solution is quite noticeably higher than that of the NIPS dope solution, consequently, the existence of more β phase in the surface layer might be related to this point.

**Table 2:** Ratio of α phase to β phase in the outer surface layer of PVDF membranes by different preparation methods.

Membrane label	α/β
NIPS membrane	1.30
Melt spinning membrane	0.88
F5-60	0.33
F5-140	1.10
F5-7-60	0.56
F5-7-140	1.14

As exhibited in Figure 2 and Table 2, there is a great difference in the characteristic bands of α and β phases of the solution-cast membranes prepared under different conditions. When the evaporation temperature is lower (at 60 °C), the characteristic bands of the α phase become much weaker, and the characteristic bands of the β phase become stronger. As reported, in the solution-cast membranes it seemed that the resulted crystalline phase of the surface was determined by the crystallization rate [14,23]. An explanation for this phenomenon might be given considering that the β phase in PVDF is thermodynamically meta-stable, however, the α phase is thermodynamically more stable. When the annealing temperature is 60 °C, the crystallization rate is slow, so that the PVDF chains can relax and have sufficient time and thermal energy to form the thermodynamically meta-stable β phase. In contrast, when the membranes are evaporated at 140 °C, which is higher than the crystallizing point 138 °C, the mobility of chains is improved a lot and the degree of the entanglemen of the chains becomes lower, which inhibits the formation of the β phase, consequently, the α/β value increases greatly.

As shown in Figure 2 and Table 2, PG as a self-made additive also exerts an influence on the α/β values: the characteristic bands of the α phase at 764 cm^−1^ and 976 cm^−1^ of the F5-7-60 membrane are much stronger than for the F5-60 membrane; the characteristic band of the α phase at 796 cm^−1^ of the F5-7-140 membrane is stronger than for the F5-140 membrane, the characteristic band of the β phase at 840 cm^−1^ of the F5-7-140 membrane is weaker than for the F5-140 membrane. The addition of PG makes the characteristic bands of the α phase become stronger, and the β phase tends to be weaker. PG as a self-made additive has a good solubility in the dope solution. The content of solvent decreases with the addition of PG, which may reduce the time of the evaporation process, consequently, the crystallization rate is enhanced, and the α/β value increases slightly.

### 3.2. Crystalline Structures of the Overall Membranes

**Figure 3:**
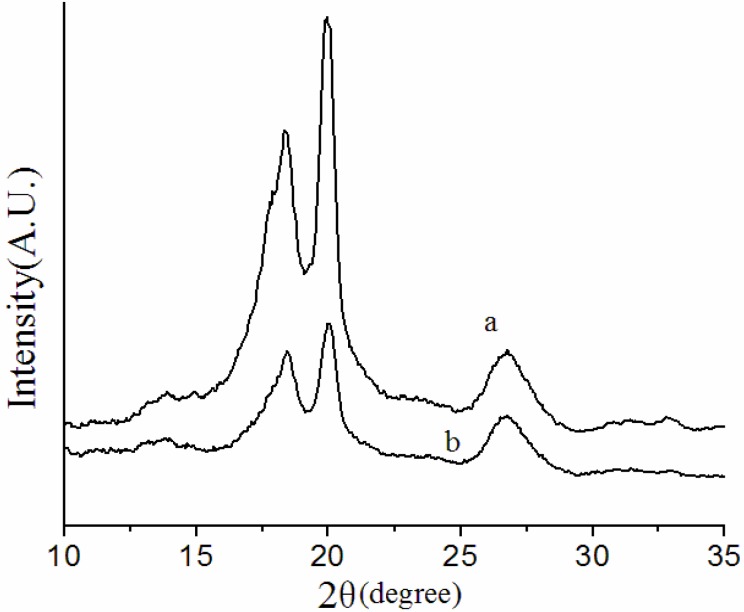
X-ray diffraction (XRD) patterns of (**a**) NIPS membrane; (**b**) melt spinning membrane.

**Figure 4:**
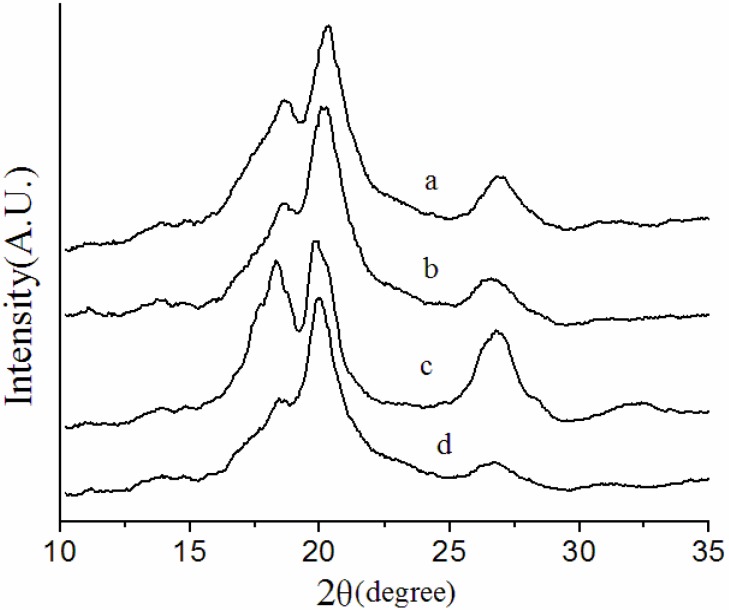
XRD patterns of solution-cast membranes prepared at different conditions. (**a**) F5-60; (**b**) F5-7-60; (**c**) F5-140; (**d**) F5-7-140.

X-ray diffraction (XRD) measurements were performed to examine the crystalline phases of the overall membranes. Figure 3 and Figure 4 show the XRD patterns of the NIPS membrane, melt spinning membrane and solution-cast membranes prepared at different conditions.

As illustrated in Figure 3, the α phase is definitely predominantly PVDF crystalline with three most distinctive diffraction peaks at around 2θ = 18.3°, 19.9° and 26.7° in the XRD patterns of NIPS and melt spinning membranes. In the preparation of the NIPS membrane, the dope solution extruded out of the spinneret was immersed into 50 °C water bath after a 3 cm air bath. As the casting solution was immersed into water bath, mass exchange between solvent and non-solvent was so fast that liquid-liquid demixing took place immediately [14,15,16]. The coagulation bath at 50 °C accelerated the solvent withdrawal process, consequently, the crystallization rate, resulted predominantly in the α phase. In the melt spinning process, the casting solution extruded out at 230 °C was immersed into a 25 °C water bath after a 50 cm air bath. Although the longer air bath could have slowed down the curing rate of the outer surface, the drawing rate was too fast, melt spinning membranes were quenched in a 25 °C water bath immediately, resulting in a high crystallization rate and, consequently, with predominant formation of the α phase in the overall membrane. These results demonstrate again that it is the crystallization rate that determines the crystalline phase of PVDF. The crystallinity of a crystalline polymer can be related to the total scattering height of the peak in the XRD spectra [32]. Thus, the difference between NIPS and melt spinning XRD patterns might be due to the difference in total crystallinity of the two kinds of membranes prepared by different methods.

It can be clearly seen from Figure 4 that the evaporation temperature exerts a large effect on the crystallization behavior of the solution-cast membranes. The membranes evaporated at 60 °C have a significant diffraction peak of β phase at 2θ = 20.6°, while the membranes evaporated at 140 °C are predominantly of the α phase with three significant diffraction peaks at 2θ = 18.3°, 19.9° and 26.7°. The disappearance of characteristic diffraction peaks of the β phase of the membranes evaporated at 140 °C might be related to the higher crystallization rate, as discussed above for the FTIR-ATR spectra. The characteristic peaks of α phase at 2θ = 18.3° and 26.7° of the solution-cast membranes with PG evaporated at 60 and 140 °C become weaker, this change might be due to the change in total crystallinity of the solution-cast membranes.

### 3.3. Thermal Behavior of the Membranes

**Figure 5:**
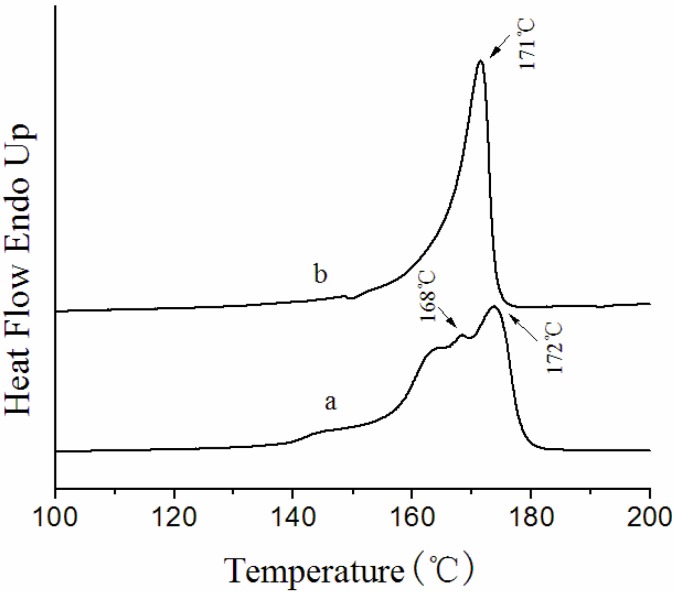
Differential scanning calorimetry (DSC) curves of (**a**) NIPS membrane; (**b**) melt spinning membrane.

Figure 5 shows the DSC curves of NIPS and the melt spinning membranes. Melt spinning membranes have one broad melting peak at 171 °C, while in NIPS membranes two endotherms are obvious with peaks at approximately 168 and 172 °C. The heat of fusion (*∆H_f_*) and total crystallinity (*X_c_*) of NIPS and the melt spinning PVDF membranes is summarized in Table 3.

**Table 3:** Heat of fusion (∆*H_f_*) and total crystallinity (*X_c_*) of PVDF membranes by different preparation methods.

Membrane label	*∆H_f_*(J/g)	*X_c_*(%)
NIPS membrane	62.8	60.0
Melt spinning membrane	51.0	48.7
F5-60	73.3	70.0
F5-140	66.0	63.0
F5-7-60	65.8	62.8
F5-7-140	57.9	55.3

A double endotherm can be usually found in the DSC curve of semi-crystalline polymers. There are mainly two interpretations for this phenomenon [33]. One explanation is that the double endotherm is due to the melting of two different crystalline phases coexisting initially. The other is that the lower-temperature endotherm does not correspond to complete melting of a crystalline phase, but rather to melting of the imperfect crystalline region or a solid-solid phase transition. The higher-temperature endotherm is associated with melting of the crystalline phase formed by such transitions as orientation changes of crystals or re-crystallization.

It had been verified that the surface layer and overall membranes had a prevalent existence of the α phase based on our FTIR-ATR and XRD results. Therefore, in this case it is likely to be the level of crystal perfection rather than the different crystalline phases that determines the melting endotherms [21]. The two endotherms in the NIPS membrane present an α phase crystalline structure with different crystal perfections. There is only one sharp endotherm with a peak at 171 °C in the DSC curve of the melt spinning membrane, which is probably due to the melting of larger and well-formed folded-chain crystals formed from a melt crystallization at 230 °C.

It can be seen from Table 3 that the *X_c_* of the NIPS membrane is much higher than for the melt spinning membrane. The reason for this change can be interpreted by considering that in the preparation of the melt spinning membrane, the as-spun hollow fiber membranes extruded out of the spinneret at 230 °C were quenched in a 25 °C water bath. As the cooling rate is too fast, the chains’ movement cannot comply with the temperature changes. Thereby the PVDF hollow fiber membranes crystallize at lower temperature, the PVDF chains have poor activity, and the crystals grow slowly, consequently, *X_c_* decreases.

**Figure 6:**
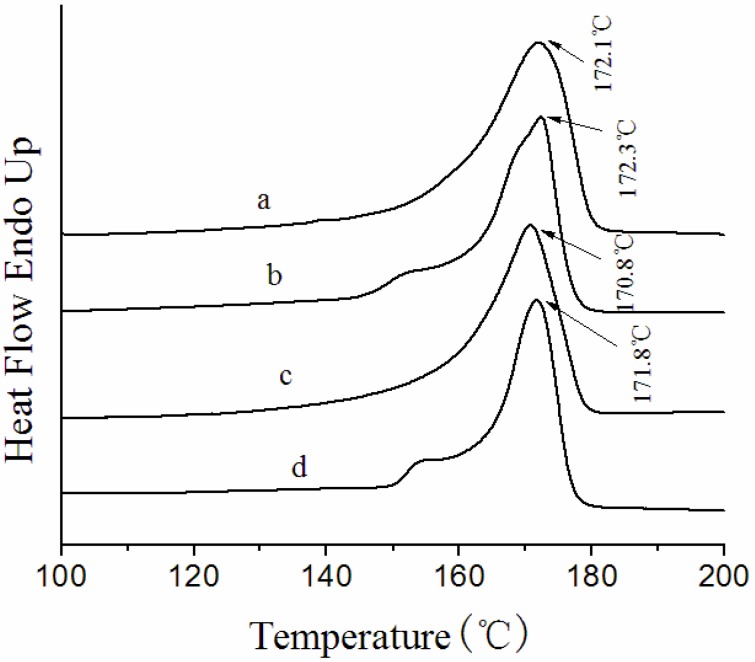
DSC curves of solution-cast membranes prepared under different conditions. (**a**) F5-60; (**b**) F5-140; (**c**) F5-7-60; (**d**) F5-7-140.

Figure 6 shows typical DSC curves of solution-cast membranes prepared under different conditions. The DSC curves present very similar thermograms, they all have one endotherm with peaks at around 170–173 °C. The heat of fusion (*∆H_f_*) and total crystallinity (*X_c_*) of the solution-cast membranes is listed in Table 3.

The melting temperatures of the solution-cast membranes evaporated at 140 °C are slightly higher than that of membranes evaporated at 60 °C. This change is predictable, when the evaporation temperature is 140 °C, which is higher than the crystallizing point 138 °C, both precipitation crystallization and melt crystallization contribute to the formation of more perfect crystalline structure, thus, T_m_ increases for the crystalline phase. As reported by Nasir *et al*. [34], the increase of the β phase could also induce a decline of the melting temperature of PVDF.

In addition, it is obvious that the addition of PG also has slight effect on the melting temperature. The melting temperature becomes a little lower, when PG is added into the casting solution. This change is probably because plasticizers and soluble additives are often added in polymer processing, which can induce a decrease in the melting temperature of the polymer; this phenomenon is called dilution effect. PG has a good solubility in the casting solution, consequently, the addition of PG results in a slight decrease in melting temperature.

As can be seen in Table 3, the *X_c_* of solution-cast membranes evaporated at 60 °C is much higher than that of membranes evaporated at 140 °C. When the evaporation temperature is 60 °C, solvent is evaporated at a slower evaporation rate, which means there is more time for the membranes to crystallize, consequently, *X_c_* is greatly improved. It can be clearly seen that the addition of PG also exerts an influence on the *X_c_* of the solution-cast membranes. The *X_c_* of solution-cast membranes becomes much lower when PG is added into the casting solution. It is the solubility in the solvent that determines whether an additive can become a nucleating agent. A soluble additive can be regarded as a diluent agent, which can inhibit the formation of a crystal nucleus. The number of crystal nuclei decrease, consequently, the total crystallinity of the PVDF decreases.

### 3.4. Crystalline Morphology

**Figure 7:**
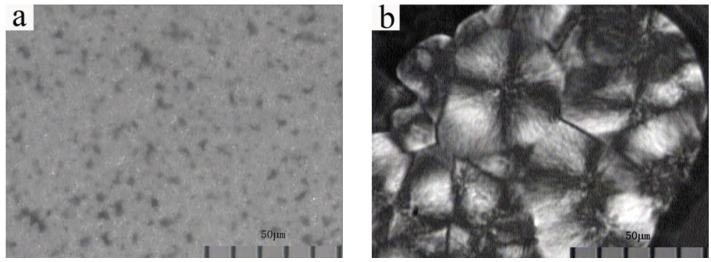
Polarized light optical micrograph of (**a**) NIPS membrane; (**b**) melt spinning membrane.

Figure 7 shows a polarized light optical micrograph of NIPS and melt spinning membranes. The NIPS membrane presents no Maltese-cross pattern. On the contrary, the melt spinning membrane shows perfect spherulites with diameters between 20 and 40 µm, which present polygons extruded by a plurality of spherulites with a clear interface. It is because of the homogeneous nucleation that the PVDF chains themselves can form crystal nuclei to induce melt crystallization in the melting process of the polymer. This nucleation process usually generates less crystal nuclei, and the shape of spherulites is generally incomplete, induced by collisions of adjacent growing spherulites.

**Figure 8:**
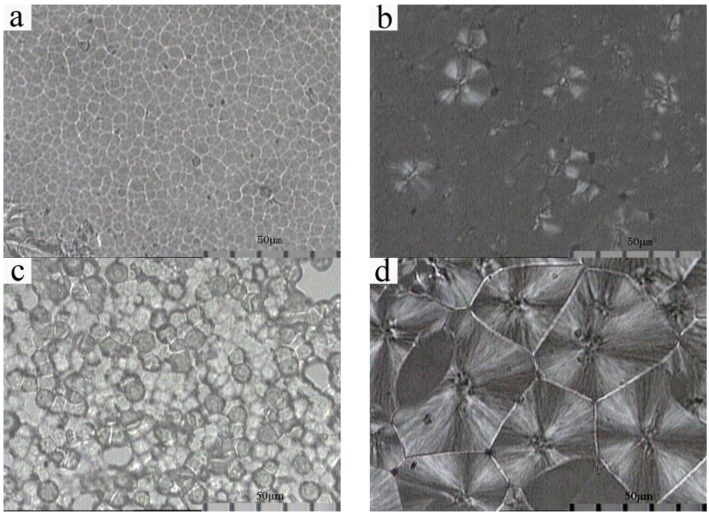
Polarized light optical micrograph of solution-cast membranes prepared at different conditions. (**a**) F5-60; (**b**) F5-140; (**c**) F5-7-60; (**d**) F5-7-140.

As can be seen in Figure 8, solution-cast membranes prepared at different fabrication conditions present significantly different crystalline structures. F5-60 membrane shows spherical particles with diameter between 6 and 8 µm, which presents no Maltese-cross pattern; F5-140 membrane shows lamellar particles with diameter between 25 and 35 µm, which presents a Maltese-cross pattern in the area with a larger phase dimension; F5-7-60 membrane shows loose piled spherical particles with diameter between 9 and 13 µm, which exhibits no Maltese-cross pattern; F5-7-140 membrane shows a Maltese-cross pattern of spherulites with diameter between 50 and 60 µm. The phase dimension of the solution-cast membranes with PG evaporated at both 60 and 140 °C become larger, and the solution-cast membranes evaporated at 140 °C present a Maltese-cross pattern.

An explanation for this difference in crystalline structure might be given considering the following: when the evaporation temperature is 60 °C, PVDF chains move slowly, and the crystallization rate is slow. On the contrary, as the membranes are evaporated at 140 °C, which is higher than the crystallizing point 138 °C, both precipitation crystallization and melt crystallization contribute to the formation of a more perfect crystalline phase, and the crystallization temperature is high enough to ensure sufficiently fast transport of macromolecules to the growth front of spherulites, consequently, the formation of spherulites has a close relationship with the evaporation temperature. PG with a good solubility in dope solution can induce a dilution effect, which might inhibit the formation of a crystal nucleus. When the evaporation temperature is 140 °C, PVDF chains move more actively, radial growth of spherulites can occur from finite crystal nucleus. Therefore, F5-7-140 membranes present obviously larger spherulites with a more perfect crystal structure.

### 3.5. Tensile Strength of the Membranes

**Figure 9:**
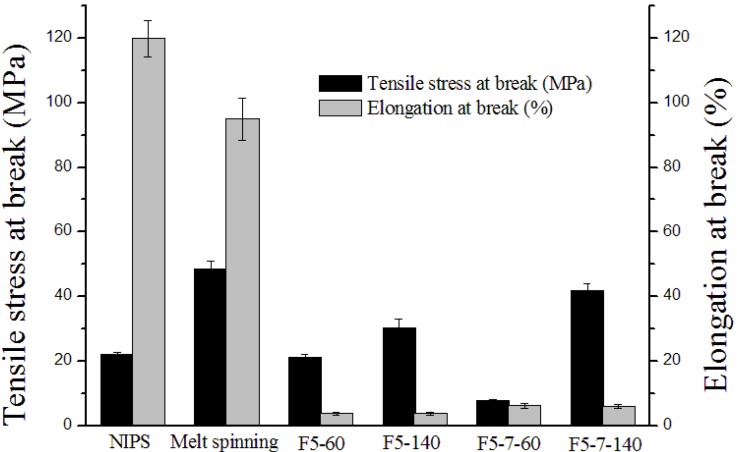
Mechanical properties of PVDF membranes with different preparation methods.

As shown in Figure 9, the preparation methods exert a great influence on the tensile strength of the membrane. The tensile strengths of the melt spinning membrane, F5-140 and F5-7-140 membranes are significantly higher than for the NIPS membrane, F5-60 and F5-7-60 membranes, and the tensile strength of melt spinning membrane reaches a maximum.

The current work investigated the differences in tensile strengths of PVDF membranes prepared by various methods mainly from several of the following aspects: crystal form, crystallinity, and crystalline morphology. NIPS membrane, melt spinning membrane and solution-cast membrane all had a mixture of α and β phases in the skin; and the α phase was definitely predominantly PVDF crystalline in NIPS and melt spinning PVDF membranes. The membranes prepared by different methods had the same crystal form, hence, it was not the crystal form that affected the tensile strength. As to crystallinity, the *X_c_* of the NIPS membrane (60.0%) was much higher than the melt spinning membrane (48.7%), however, the tensile strengths had the opposite trend, which was contrary to common sense that the higher the crystallinity, the higher the tensile strength. This result illustrated that crystallinity was not the critical factor that influenced tensile strength. As discussed in crystalline morphology, the NIPS membrane, F5-60 and F5-7-60 membranes presented no Maltese-cross pattern, however, melt spinning membrane and the F5-7-140 membrane showed obvious spherulites with a Maltese-cross pattern, the tensile strengths of membranes with spherulites were much higher, consequently, crystalline morphology was considered to be the main factor that influences the tensile strength of the membrane.

The mechanical behavior of PVDF membranes is closely linked to crystalline morphology, which depends strongly on the crystallization process. Different crystalline morphologies lead to great differences in the crystalline phase and the amorphous-crystalline interfacial region.

As reported by Wallner *et al*. [35], the fracture process of the α phase PVDF film was accompanied by breakdown of the spherulitic structure to a fiber bundle structure. Hence, the formation process of spherulite is referred to, to study the effect of the crystalline phase on the tensile strength. Crystallites begin to grow from a single nucleus, and form ribbon-like lamellae (fibrils). Then the continuous growth of the lamellae can twist, twin (form two or more branches), or bisect and results in the formation of spherulites [31]. The fracture of semi-crystalline polymer with a spherulite structure starts with the interfacial failure of the spherulitic superstructure. Furthermore, the spherulites are ruptured to give an orientated fibrous structure [36]. The deformation of spherulites is always accompanied by great stress, consequently, PVDF membranes with a spherulitic structure (melt spinning membrane, F5-140 and F5-7-140 membranes) have higher tensile strength.

For NIPS membrane, F5-60 and F5-7-60 membranes, these PVDF crystallites do not stack compactly and have an obvious spherulitic shape with a Maltese-cross pattern. Hence, there are more crystals but of smaller size. This increases the interfacial region and reduces the degree of order of the system, so the tensile strength is lower.

## 4. Conclusions

In this study, PVDF membranes were prepared through NIPS, melt spinning and a solution-cast method. The effect of the preparation methods on the crystallization behavior and tensile strength of PVDF membranes was investigated. The main conclusions from the experiments are summarized as follows:

(1) The NIPS PVDF membrane had a mixture of α and β phases in the outer skin and was predominantly of α phase in the overall membrane, total crystallinity was 60.0% with no spherulite;
(2) The melt spinning PVDF membrane had a mixture of α and β phases in the outer skin and was predominantly of α phase in the overall membrane, total crystallinity was 48.7% with perfect spherulites;
(3) The crystallization behavior of the solution-cast membranes was related to the evaporation temperature and additive: when the evaporation temperature was lower (at 60 °C), total crystallinity increased and PVDF formed the β phase more favorably; As the soluble additive was added into the dope solution, the total crystallinity decreased, the α phase in the outer skin increased, and the crystal conformation of the overall membranes had no significant change. When the evaporation temperature was 140 °C with a soluble additive in the dope solution, obvious spherulites were observed;
(4) Among the PVDF membranes prepared by various methods, it was crystalline morphology rather than crystal form and total crystallinity that determined the tensile strength. The difference in crystalline phase and amorphous-crystalline interfacial region induced by different crystalline morphologies exerted a great influence on the tensile strengths of the membranes, which were much higher with perfect spherulites.
